# A virtual global carbon price is essential to drive rapid decarbonisation

**DOI:** 10.14324/111.444/ucloe.1983

**Published:** 2024-12-17

**Authors:** Richard H. Clarke, Mark A. Maslin

**Affiliations:** 1 Ortec Finance, Bridge House, 181 Queen Victoria Street, London EC4V 4EG, UK; 2 University College London, Gower Street, London WC1E 6BT, UK

**Keywords:** carbon, climate change, carbon price, net zero, engineering, loss and damage

## Abstract

Dealing with climate change is now an infrastructure challenge. Within the next 30 years our energy generation must switch from fossil fuels to renewables. New buildings need to be zero-carbon and existing buildings need to be retrofitted. Our global transportation network will need to be transformed. Delivering the Net Zero World is an engineering challenge. But to do this we need a globally agreed virtual carbon price so that every single infrastructure project can be assessed in terms of its impact on carbon emissions and thus planetary health. We propose a loss-and-damage-based carbon price that is enhanced or reduced by variable, national impact factors. Carbon intensity weighting would further increase the price’s impact.

## Introduction

The behaviours of engineers are triangulated by the needs of their employer, their education, training, experience, character and the guidance and rules of their professional bodies. Martin [1] highlights that leading employers and leaders of the engineering community are aware of the need for the profession to change its approach to infrastructure in the face of the challenges of a changing climate. While some employers are far-sighted and holistic, many are not. So, it is incumbent on the professional bodies to be the guardians of public wellbeing, safety and the environment.

Much change has been achieved by the engineering profession in recent decades. Safety engineering has become its own discipline. Energy efficiency, resource utilisation, local pollution abatement and cost reductions have enabled mass access to transport, technology and cheap food. But some of this has been done at the expense of the global environment. A more holistic approach to ‘safety’ in its broadest sense is required, to deal with global issues such as greenhouse gas (GHG) emissions and plastic pollution. Total lifecycle thinking must become the norm for all engineers and project developers [2].

For example, if a power plant were to be built today, and Net Zero 2050 is the target, then it would, in theory, need to emit less than half as much carbon dioxide (CO_2_) as a plant commissioned 40 years ago. If this cannot be done, or is uneconomic, then, with current approaches, the project must be justified by energy policy or subsidised or both. These approaches cause engineers to deliver unsustainable projects in the face of conflicting influences from international treaties, insurers and pressure from the law and some investor and societal groups. Engineers, and, indeed, all these groups need a common tool to encourage the design and delivery of infrastructure projects that are consistent with net zero ambitions. We propose that a virtual weighted carbon price based on the carbon intensity and consequent climate change damages could be used as one such a tool to help track progress to net zero at the national scale that includes some adjustments to compensate for historical emissions.

## Methods

### Calculating the carbon intensity weighting

**Figure 1:**
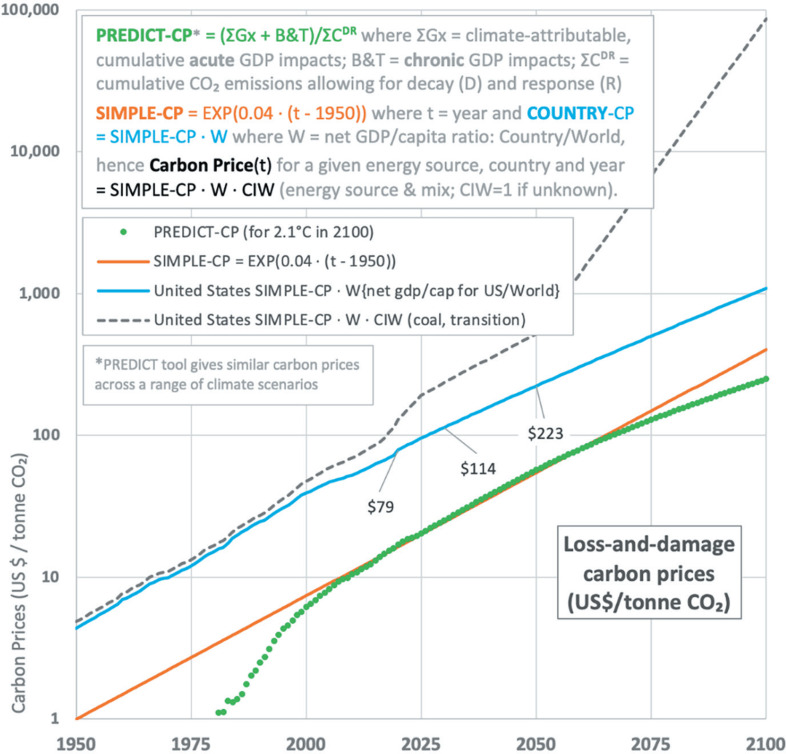
The cumulative, climate change related economic impacts of carbon emissions has escalated since the 1980s (green/orange lines) and continued ‘business as usual’ (2.6 °C in 2100) emissions are expected to lead to catastrophic losses, especially in low- and middle-income (LMIC) countries. The PREDICT-CP carbon price (green line) captures the modelled, global GDP impacts of acute physical risk (extreme weather) and chronic physical risk in 154 countries (using aggregates of 1860 city-based polygons; we note that about a third of all disasters occur within the boundaries of cities). These historical and future GDP impacts were calculated using the Ortec Finance PREDICT tool. PREDICT shows that the impact of acute risk under RCP8.5 (4.3 °C of warming by 2100) could cause a difference-to-baseline reduction in global GDP of about 60% by 2100. This is similar to Kotz et al. [4]. The underlying data comes from World Urbanization Prospects (WUP, United Nations, New York), NOAA annual temperature anomalies, historical/projected temperature anomaly trends by country (NASA-GISS) and Munich Re/EM-DAT (disaster and catastrophe frequencies and losses, by location and peril, 1980–2018).

In this section we propose how to calculate the carbon intensity of the energy sources involved in any infrastructure project. Then we set out how this can be incorporated into a virtual carbon price and how a weighted carbon price can be used to track progress towards net zero at the scale of nations. We use this approach because there is a particular problem with carbon pricing as it can be a one-size-fits-all, making carbon price a blunt instrument for encouraging behavioural change. A spectrum of prices based on impact (carbon intensity) would be more effective as well as future-proof [3]. For a carbon price to be credible it must provide a sustained signal of significant magnitude, one that is both verifiable and reasonably predictable. This, we believe, is where our loss-and-damage-based carbon price (Fig. 1) has an advantage.

Two things then become apparent. Firstly, to incentivise the movement from ‘dirty’ carbon-intensive fuels to ‘clean’ low-carbon fuels or energy, there may need to be an even stronger price signal, whatever the base price. Secondly, to ensure continuing best practice it will be necessary, from the very start, to link the carbon prices to all energy types and not just fossil fuels.

For every fuel or energy source there is a ratio e, the amount of CO_2_ emitted divided by the useful energy the source produces. This is called ‘carbon intensity’. For coal, e is about 1 tonne/MWh of electricity; for gas it is about 0.46 tonne/MWh, but even with renewable energy and nuclear sources there is a hidden e of between 0.01 and 0.05 tonne/MWh due to their materials of construction. We use this information to create a carbon intensity weighting (CIW).

By using the CIW method, the carbon price y_i_ for fuel/energy type i is given by

$${\text{y}}_{\text{i}}=\text{y}\times \text{CIW}=\text{y}\times {\text{e}}_{\text{i}}\times \text{f}\times \text{z}.$$

The ‘CIW’ factor f is defined as

$$\text{f}=\Sigma {\text{E}}_{\text{i}}\text{/}\Sigma {(\text{E}}_{\text{i}}\times {\text{e}}_{\text{i}})\text{.}$$

**Figure 2:**
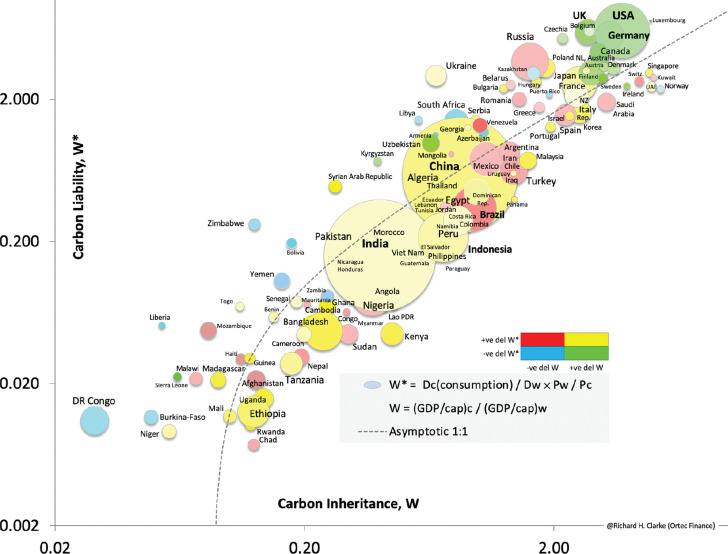
GDP - consumption emissions plot: (D_c_/D_w_ × P_w_/P_c_) v. (GDP/capita)_c_/(GDP/capita)_w_ at time t, where D_c_ = country (consumption) cumulative emissions, D_w_ = world cumulative emissions, P_w_ = world population, P_c_ = country population. The effective country weighting, W_eff_ is (W × W*)^0.5^, where W is the carbon inheritance and W* is the carbon liability. If only GDP/capita data is available, set W_eff_ = W and if country weightings are not required, set W_eff_ = 1. The bubbles are coloured according to the colour key: for example, if a country’s W decreases and W* increases, the bubble will be a shade of red. The data behind this figure comes from sources quoted in Fig. 1 and population, GDP per capita and granular emissions data by territory are compiled and curated by Our World in Data (OWiD, Oxford). The diagram uses, where available, the cumulative consumption emissions from 1750 to 2017; the consumption emissions of nations include emissions associated with imported goods and services. Bubble colours reflect the changes from 2016 to 2017.

A ‘revenue weighting’ factor z is defined as the weighting needed to ensure that the total premium from individual fuel prices y_i_ is consistent with the premium using a global, unadjusted carbon price y.

$$\text{z}={(\Sigma ({\text{E}}_{\text{i}}\times {\text{e}}_{\text{i}}))}^{2}\text{/(}\Sigma {\text{E}}_{\text{i}}\times \Sigma ({\text{E}}_{\text{i}}\times {\text{e}}_{\text{i}}^{2})\text{),}$$

where,

E_i_ = amount of fuel/energy type i used globally (or by country or sector or, perhaps, by company) (GWh)

e_i_ = emission factor for fuel/energy type i (tonne CO_2_/GWh)

y_i_ = carbon price for a given fuel/energy type i (US$/tonne CO_2_)

y = global carbon price (US$/tonne CO_2_), for example, y = SIMPLE-CP × W_eff_ (see main text and Figs. 1 and 2).

### Calculating the impact of CO_2_ decay and climatic response

The peak impact from injecting a mass of CO_2_ into the atmosphere occurs about 20 years after its release. We calculate the impact of cumulative, global emissions ΣC^DR^ using a two-step approximation.

1. Decay. The estimated lifetime of a mass of fossil CO_2_ in the atmosphere is calculated using a fit to the ensemble predictions reported by Archer et al. [5]. From the year of its release, t_i_, to a future year, t_n_, the proportion, C*, of the initial release, C, that remains airborne is given by:
$$\text{C* = C}\times \text{(0.22+0}{\text{.27e}}^{-(t_{\text{n}}-t_{\text{i}})/350}+0.35{\text{e}}^{-(t_{\text{n}}-t_{\text{i}})/200}+0.16{\text{e}}^{-(t_{\text{n}}-t_{\text{i}})/10}).$$
2. Response. The fractional surface temperature response R to a doubling of atmospheric CO_2_ is initially fast (~40% in 8 years) but then levels off. According to Hansen et al. [6], equilibrium may take over 1000 years to be reached, largely due to the oceans. Roper approximated this (http://roperld.com/science/GlobalWarmingPrediction.htm) using a two-term equation:
$$\text{R}=0.368\times \text{tanh}(({\text{t}}_{\text{n}}-{\text{t}}_{\text{i}})\text{/}10.5)+0.632\text{/}2\times (1+\text{tanh}(({\text{t}}_{\text{n}}-{\text{t}}_{\text{i}}-277)\text{/}524)).$$

Combining C*, R and historical emissions data (Our World in Data) in a matrix calculation yields the decay and response adjusted, cumulative emissions data ΣC^DR^ that is needed to determine the cumulative carbon price PREDICT-CP (see Fig. 1). For the years in which t_n_ < t_i_ the matrix contains zeroes. Historically, ΣC^DR^ ≈ 0.368 × ΣC.

## Carbon pricing for engineers

An alternative approach to policies or subsidies is to address the loss and damage caused by CO_2_ specifically. We argue there needs to be an internationally agreed, virtual carbon pricing system that can readily be used by engineers to estimate the economic impact of each tonne of CO_2_ or any other GHG emitted (Fig. 1). Those costs should be included in the economic assessment of every project [7]. When and where a project takes place are significant factors.

Carbon markets are unpredictable, and other carbon pricing tools are complex to use, or they are encumbered by social discounting considerations [8]. An engineer always needs a practical equation. We propose that a loss-and-damage-based carbon price is used in all projects where carbon or GHG emissions occur. This would include direct and embodied emissions, for example, steel or concrete.

In Fig. 1 the base carbon price (SIMPLE-CP, orange line) represents the carbon price that would compensate for the cumulative, climate attributable economic impact (G*x*) of cumulative CO_2_ emissions (ΣC^DR^); these are summated global emissions C adjusted for decay and climatic response (see Methods section). G is the economic damage from acute physical risks (extreme weather) and *x* is the extent to which those losses are climate attributable. Here, the attribution factor is determined using a proxy based on local temperature anomaly.

The simplified carbon price, SIMPLE-CP (US$, 2020) = e^(0.04 × (year-1950))^ is an approximation to the output of Ortec Finance’s PREDICT physical risk tool, as modified to produce the loss-and-damage carbon price PREDICT-CP (see Fig. 1 for details). For 2025, the SIMPLE-CP = US$20/tonne CO_2_. The B&T (Burke and Tanutama) term (Fig. 1), accounts for the economic damage from chronic or slow-onset physical risks [9]. The base carbon price is largely independent of future emissions, provided that the transient climate response to cumulative emissions (TCRE) holds at about 1.9 °C/trillion tonnes carbon. This base price is then factored by a time-varying, country weighting factor (W_eff_, or W for simplicity, see Fig. 2) as the historic emissions and their associated economic development should be considered, to address the need for climate justice [10]. By including W, the United States (US) country price would be $100 in 2025. Additionally, a CIW term can be included to address laggard, high carbon intensity emissions (see Methods section). Thus, the loss and damage carbon price (for year, country, fuel/energy type) = SIMPLE-CP × W × CIW.

As an example, coal emissions in the US in 2030 would attract a carbon price of over $272/tonne CO_2_ = US$ e^0.04 × (2030-1950)^ × 5.35 × 2.07. The CIW term depends on the future energy mix and geographical or sectorial scope (Clarke [3] showed how CIW could evolve during an energy transition). This price is robustly in line with the proposals of the World Bank Carbon Pricing Leadership Coalition’s High-Level Commission. By mid-century, the impacts of acute and chronic physical risk are about equal. Callaghan and Mankin [11] showed the profound impact that chronic physical risk is already causing. The country weighting factors, W, include the effects of chronic physical risk.

## Prioritising infrastructure changes in the Developed World first

The engineering challenge of net zero is even harder when it is realised that not even the richest countries have truly started to decouple their energy use from emissions [12]. The terms carbon inheritance and carbon liability convey the immutable relationship between economic wealth (gross domestic product [GDP]/capita) and energy (kWh/GDP) see Webster and Clarke [13].

We define carbon inheritance (W) as the wealth that nations have attained, largely by using fossil fuels since the beginning of the Industrial Revolution or as data permits. More specifically, this inheritance relates to work and energy but, in practice, nearly all that energy has come from fossil energy. W is expressed as the ratio of (GDP/capita)_country_/(GDP/capita)_world_, so the exact definition of GDP is immaterial.

The second term, carbon liability (W*), we define as the cumulative carbon emissions D (= ΣC) of a country divided by its current population (D_c_/P_c_) and the result is then divided by (D_world_/P_world_). We argue that the current populations represent the net outcome of all the progress, toil, conflict, health and other factors that have led to the emissions and wealth of a country today.

Overall, we find there is a direct relationship (R^2^ = 0.63) between cumulative wealth and cumulative emissions, as shown in Fig. 2. For each country, the emissions and wealth have been normalised using the global average values as noted above. The size of the bubbles is proportional to the current population of each nation. On the log–log plot there is roughly a 1:1 relationship between scaled emissions and scaled GDP, with a few outliers. The relationship is strongest if consumption, rather than domestic-only emissions are included.

There is a huge difference between the Democratic Republic of the Congo and the US, over two orders of magnitude in fact. This is because the USA has inherited a lot of emissions from its own systems and has a lot of liability as well which is the opposite for the DR Congo. Figure 2 makes a compelling case for action by the industrialised, first-tier economies. When their populations are factored-in, the impact of US, China, Japan, Germany, United Kingdom (UK) and other high-income countries becomes apparent. Whatever else they do, these countries need to fully commit to net zero, and allow engineers to lead the infrastructure revolution, to enable the energy transition. The benefits to these countries and all the others would be transformational. To take a specific example, the UK is blessed with copious quantities of offshore and onshore wind and yet the previous UK Government committed to yet more North Sea oil production and that may not pass the net zero tests, as determined by the UK Government’s own Committee on Climate Change [14]. Rather, the UK should lead on the seasonal energy storage technologies and inter-country grid connectors that are needed to make a renewables-dominated grid dependable. Moreover, there are too many instances in which the UK Government has been taken to court due to non-compliance with legislation it previously enacted, for example, in meeting its 2030 targets or poor home insulation uptake. Currently, the developing economies and India, in particular, look to the UK for leadership as one of the founders of the industrial age.

The underlying data behind Fig. 2 includes population, GDP data and all-forms of emissions data and these can be regularly updated. This leads to the possibility that the diagram could be used as a tool for tracking the progress of nations towards net zero.

For example, if a nation’s bubble moves:

Horizontally right – the economy is growing faster than the global average with low emissions (good, a shade of green).

Right and up – that is, ‘business as usual’ growth (must do better, a shade of yellow).

Stands still – in line with global average (fair, yellow).

Left and down – economy is in trouble (blue, policy action needed).

Up and left, pink as per Brazil or red as per Venezuela (deep trouble, emigration, possible economic collapse).

Right and down – has Sweden started transitioning as its population grows? (good, a deeper shade of green).

### Discussion of actions to drive net zero

The need for rapid transition to renewable energy has become central to the discussion of energy security. The Russian invasion of Ukraine led to a huge increase in fossil fuel prices which affected everything from industry, agriculture to the cost of living. In terms of infrastructure, a mixed response is emerging: the European Union is moving away from Russian gas as quickly as possible, having pledged to double the installation of renewable energy this decade [15]; meanwhile, in the US the Biden administration opened the door to selling new oil and gas drilling leases in the Gulf of Mexico and Alaska to help it ensure self-sufficiency in fossil fuels. It has proposed as many as 11 lease sales over the next five years, including 10 in the Gulf of Mexico and one in the Cook Inlet off the Alaskan coast [16]. Drilling, however, off both the Atlantic and Pacific coasts are not included. Meanwhile China, and to a lesser extent India, have leapt at the opportunity to buy cheap Russian oil, due to Western sanctions on Russian exports. Imports of Russian oil rose by 55% from a year earlier to a record level in May 2022, displacing Saudi Arabia as China’s biggest provider [17].

Longer term, the invasion of Ukraine has put energy security back on the top of governments’ agendas. For countries with no or little access to domestic fossil fuel reserves, renewables are set to become very attractive – they are already cheaper to build and maintain than coal fired power stations (International Energy Agency). Hence a diagram such as Fig. 2 will enable us to track how countries are doing not only in decarbonisation but also how secure their energy will be in the future.

As well as an agreed virtual carbon price, professional bodies need to dissuade companies and individuals from the defensive patenting of clean technologies and should instead support licensing agreements to ensure that smart ideas reach the market. This will give a clear signal to incumbents that they need to transition their technologies or move to new markets. As the Carbon Disclosure Project [18] highlights, it is policy and attitude as well as low emissions that makes for a clean, net zero-aligned corporation. On every board and division, there needs to be an executive level officer who is responsible for transition compliance and lifecycle engineering.

Thus, to empower engineers and to kick-start or boost the net zero revolution in the developed markets followed by the rapidly emerging markets, we call for four actions:

1. Engineering professional bodies across the world need to support engineers so they are empowered to do the job they need to do, to enable economies to rapidly decarbonise their energy, infrastructure, manufacturing and food industries.
2. Every major company needs a Net Zero Transition Compliance Officer who alongside the Safety Compliance officer ensures every project and decision helps develop the green, low-carbon economy.
3. Develop the carbon inheritance/carbon liability diagram (Fig. 2) to monitor the movements of countries, to determine if and to what extent they are on track during the energy transition. Ideally, the clock rate on this should be faster than once per year.
4. Establish a usable yet meaningful globally agreed virtual carbon price, together with carbon auditing tools [19] so that engineers and other actors can include the cost of emitting each tonne of CO_2_ in determining the economic feasibility of projects. A method is suggested above but, ideally, all engineers in the world need to be using the same tool to check that every infrastructure project complies with the Paris Agreement decarbonisation pathway.

A huge side benefit of all this will be to draw the world’s exceptionally talented individuals into the engineering profession, to work on holistic solutions to today’s and tomorrow’s needs.

## Data Availability

All data generated or analysed during this study are included in this published article.

## Appendix

Ortec Finance’s acute physical risk tool PREDICT has been built to provide an estimate of the GDP impact of extreme weather events for three perils under a range of climate scenarios. This output uses econometric modelling that examines the benefits and drawbacks of different societal responses to climate change, including the impacts of the energy transition and other policy measures. Ortec Finance’s modelling is combined with E3ME, the macroeconomic model developed by Cambridge Econometrics.

PREDICT is comprised of six data arrays and three modules that together calculate the expected frequency of events N for each city-based polygon area (i = 1–1860 in 154 countries) and each peril [h = 1–3, which comprise meteorological (cyclones, etc.), hydrological (flooding, etc.), climatological (heatwaves, droughts, etc)] for each year t. The three modules include:

- an urbanisation module U (equation A.1) that is influenced by city and regional population size p(t), and change rate dp/dt;
- an adaptation module A that depends on city and regional population and GDP/capita;
- a climate module ψ that amplifies the climate-counterfactual trends in extreme weather event frequency as temperature anomalies change, as each climate scenario unfolds; the temperature anomaly T_a_ used in ψ is multiplied by a country or sub-regional factor LLE that estimates how T_a_ varies with latitude and longitude (NASA-GISS data); in ψ, for each peril, there is a global parameter δ_h_ₕ;

the data arrays are: population data p_i,t_ for each city (UN World Urbanisation Prospects + projections); GDP/capita g (at country level or below); global temperature anomalies T_a_(t); T_a_ correction factors for each city-polygon LLE_i,t_; f_h,i_ baseline expected frequency of events for each peril and each city-polygon; ε_h,i_ calibration factors for each peril and each city-polygon (ε_h,i_ values tend to 1 as the model improves; Munich Re and EM-DAT data was used to calibrate the model):

(A.1)
$${\text{N}}_{\text{h,i,t}}=\varepsilon_{\text{h,i}}\;\cdot \;\text{U}\;(\text{dp/dt},\text{ p})\;\cdot \;\text{A}\;(\text{p},\text{ g})\cdot {\text{f}}_{\text{h,i}}\cdot \psi \;{\text{(T}}_{{\text{a}}_{\text{t}}}\;\cdot \;{\text{LLE}}_{\text{i,t}}|\delta_{\text{h}}).$$

The frequencies N_h,i,t_ for each city-polygon and peril in year t are converted into GDP impacts G (2020US$) using equation A.2. Each N-term is multiplied by LPE_t_, the time-variant loss-per-event (US$; additional factors are used for meteorological events); a time variant country factor CF_t_; and EAR(g), a GDP/capita-dependent Economic Amplification Factor derived from the research of Hallegatte and Hourcade [20]. For developed economies, EAR tends to 1 as GDP/capita increases.

(A.2)
$${\text{G}}_{\text{c,t}}=\Sigma_{\text{i}}\Sigma_{\text{h}}{\text{N}}_{\text{h,i,t}}\;\cdot \;{\text{LPE}}_{\text{t}}\;\cdot \;{\text{CF}}_{\text{t}}\;\cdot \;\text{EAR}\;(\text{g}).$$

## References

[1] Martin V (2021). It is time for civil engineers to have the difficult conversations to turn climate talk into climate action.

[2] Hauschild MZ, Kara S, Røpke I (2020). Absolute sustainability: challenges to life cycle engineering. CIRP Ann.

[3] Clarke RH Carbon Intensity Weighting 2016. Chapter 10. Predicting the Price of Carbon Predict Ability Limited.

[4] Kotz M, Levermann A, Wenz L (2024). The economic commitment of climate change. Nature.

[5] Archer D, Eby M, Brovkin V, Ridgwell A, Cao L, Mikolajewicz U (2009). Atmospheric lifetime of fossil fuel carbon dioxide. Annu Rev Earth Planet Sci.

[6] Hansen J, Sato M, Kharecha P, Beerling D, Masson-Delmotte V, Pagani M (2008). Target atmospheric CO_2_: where should humanity aim?. Open Atmos Sci J.

[7] Kennelly C, Berners-Lee M, Hewitt CN (2019). Hybrid life-cycle assessment for robust, best-practice carbon accounting. J Clean Prod.

[8] Pindyck RS (2019). The social cost of carbon revisited. J Environ Econ Manag.

[9] Burke M, Tanutama V (2019). Climatic constraints on aggregate economic output.

[10] Clarke RH, Wescombe NJ, Huq S, Khan M, Kramer B, Lombardi D (2023). Climate loss-and-damage funding: a mechanism to make it work. Nature.

[11] Callahan CW, Mankin JS (2022). National attribution of historical climate damages. Climatic Change.

[12] Shukla PR, Skea J, Slade R, Al Khourdajie A, van Diemen R, McCollum D, IPCC (2023). Climate Change 2022: Mitigation of Climate Change: Contribution of Working Group III to the Sixth Assessment Report of the Intergovernmental Panel on Climate Change.

[13] Webster A, Clarke R (2017). Insurance companies should collect a carbon levy. Nature.

[14] CCC (2022). Letter: climate compatibility of new oil and gas fields.

[15] Chestney N, Zinets N Russia resumes some gas flows to Germany as its forces home in on power plant in Ukraine.

[16] Newburger E Biden opens the possibility of more offshore oil drilling in the Gulf of Mexico.

[17] Chen A Russian oil supplies to China up 22% on year, close second to Saudi – data.

[18] CDP (2022). Carbon Disclosure Project.

[19] Flannery B (2022). The greenhouse gas index: a metric for greenhouse gas-intensive products.

[20] Hallegatte S, Hourcade J-C, Dumas P (2007). Why economic dynamics matter in assessing climate damages: Illustration on extreme events. Ecol Econ.

